# The GARP/MYB-related grape transcription factor AQUILO improves cold tolerance and promotes the accumulation of raffinose family oligosaccharides

**DOI:** 10.1093/jxb/ery020

**Published:** 2018-01-29

**Authors:** Xiaoming Sun, José Tomás Matus, Darren Chern Jan Wong, Zemin Wang, Fengmei Chai, Langlang Zhang, Ting Fang, Li Zhao, Yi Wang, Yuepeng Han, Qingfeng Wang, Shaohua Li, Zhenchang Liang, Haiping Xin

**Affiliations:** 1 Beijing Key Laboratory of Grape Sciences and Enology, CAS Key Laboratory of Plant Resources, Institute of Botany, Chinese Academy of Sciences, Beijing, PR China; 2 Key Laboratory of Plant Germplasm Enhancement and Specialty Agriculture, Wuhan Botanical Garden, Chinese Academy of Sciences, Wuhan, PR China; 3 Center for Research in Agricultural Genomics (CRAG) CSIC-IRTA-UAB-UB, Cerdanyola del Vallès, Barcelona, Spain; 4 Ecology and Evolution, Research School of Biology, Australian National University, Acton, ACT, Australia

**Keywords:** Abiotic stress, Chinese wild grape, cold response, galactinol synthase, grape transformation, HRS1, low temperature exotherms, raffinose, ROS

## Abstract

Grapevine (*Vitis vinifera* L.) is a widely cultivated fruit crop whose growth and productivity are greatly affected by low temperatures. On the other hand, wild *Vitis* species represent valuable genetic resources of natural stress tolerance. We have isolated and characterized a MYB-like gene encoding a putative GARP-type transcription factor from Amur grape (*V. amurensis*) designated as *VaAQUILO*. *AQUILO* (*AQ*) is induced by cold in both *V. amurensis* and *V. vinifera*, and its overexpression results in significantly improved tolerance to cold both in transgenic Arabidopsis and in Amur grape calli. In Arabidopsis, the ectopic expression of *VaAQ* increased antioxidant enzyme activities and up-regulated reactive oxygen species- (ROS) scavenging-related genes. Comparative mRNA sequencing profiling of *35S:VaAQ* Arabidopsis plants suggests that this transcription factor is related to phosphate homeostasis like their Arabidopsis closest homologues: AtHRS1 and AtHHO2. However, when a cold stress is imposed, *AQ* is tightly associated with the cold-responsive pathway and with the raffinose family oligosaccharides (RFOs), as observed by the up-regulation of galactinol synthase (*GoLS*) and raffinose synthase genes. Gene co-expression network (GCN) and *cis*-regulatory element (CRE) analyses in grapevine indicated *AQ* as potentially regulating *VvGoLS* genes. Increased RFO content was confirmed in both transgenic Arabidopsis and Amur grape calli overexpressing *VaAQ*. Taken together, our results imply that *AQ* improves cold tolerance through promoting the accumulation of osmoprotectants.

## Teaser

The stress-responsive *AQUILO* gene regulates the expression of *GoLS* genes and improves cold tolerance in association with the increase of raffinose family oligosaccharides.

## Introduction

Low temperature is a primary environmental factor that affects plant growth and development, significantly constraining the geographic distribution of plants and affecting agricultural productivity (Chinnusamy *et al.*, 2007). Plant species have evolved multiple molecular, biochemical, and physiological adaptations to maximize cold tolerance by adjusting their metabolism (Thomashow, 2010; Theocharis *et al.*, 2012). Many genes respond to cold stress at the transcriptional level and their products may either directly protect against stress or further regulate the expression of genes involved in the different steps of a cold acclimation event (Yamaguchi-Shinozaki and Shinozaki, 2006). Accordingly, the cold response is not entirely constitutive and part of it develops after the exposure to low temperatures (Theocharis *et al.*, 2012).

At present, the best-known molecular process with a role in cold acclimation is the Arabidopsis CBF cold response pathway (Chinnusamy *et al.*, 2007). C-repeat binding factors (CBFs; also known as dehydration-responsive element-binding proteins or DREBs), act as central hubs in the cold signalling network and recognize a CRT/DRE *cis*-acting regulatory element present in the promoters of many cold-regulated (*COR*) genes (Vogel *et al.*, 2005; Yamaguchi-Shinozaki and Shinozaki, 2005; Chinnusamy *et al.*, 2007). More than 100 cold-regulated genes are responsive to CBF activation, altogether promoting an increase in cold tolerance (Chinnusamy *et al.*, 2007). In addition to the CBF pathway, CBF-independent pathways have also been proven to be required for the cold stress response (Zhu *et al.*, 2004; Li *et al.*, 2017). Direct evidence exists for the activities of some cold-regulated transcription factors not participating in the CBF cold response pathway, such as AtHOS9 (Zhu *et al.*, 2004), OsMYB3R-2 (Dai *et al.*, 2007), AtZAT12, AtMYB73, and AtWRKY33 (Jia *et al.*, 2016), which suggests that transcriptional regulation plays a crucial role in cold responses as well as in the crosstalk between different signalling pathways (Fowler and Thomashow, 2002).

For grapevine (*Vitis vinifera* L.), a widely cultivated fruit crop, cold perceived during the growth cycle represents a detrimental factor affecting productivity and quality. In northern China, all grown grapevine cultivars need to be buried in soil to protect vines from extreme winter conditions. This procedure drastically increases the costs of production and decreases the survival rate of vines. Therefore, improving cold tolerance has been a major objective for most grapevine breeding programmes in regions subject to severe low winter temperatures over a long period. Amur grape (*V. amurensis*), a popular Chinese wild grape, is the most cold-hardy species in *Vitis*, which can safely overwinter without soil cover at temperatures as low as –40 °C (Fennell, 2004). This wild *Vitis* germplasm has been used for traditional breeding for several decades. However, only a few filial generations have been obtained with enhanced cold tolerance. To reveal the molecular mechanisms Amur grape possess to respond to severe low temperature, we aimed to characterize new cold-responsive genes and to elucidate their regulatory networks.

In a previous transcriptome analysis, we showed that several genes encoding putative transcription factors were induced by low temperatures in grapevine shoot apices and leaves (Xin *et al.*, 2013). Among these, we found a MYB-like gene, encoding a putative GARP-type transcription factor, as one of the most strongly induced genes. In the current study, we focus on this gene, designated as *AQUILO* (AcQUIred tolerance to LOw temperatures; after the Greek god of northern winds and winter) and studied its relationship to cold stress tolerance. The gene was studied in both *V. amurensis* and *V. vinifera* cv. ‘Muscat Hamburg’. Both *VaAQUILO* (*VaAQ*) and *VvAQUILO* (*VvAQ*) were confirmed to be cold responsive, and the overexpression of *VaAQ* in Arabidopsis and Amur grape led to enhanced tolerance to cold stress. Several downstream genes regulated by *VaAQ* in Arabidopsis were identified using RNA sequencing (RNA-Seq), many of which overlapped with recently predicted targets of its Arabidopsis orthologues AtHRS1 (Hypersensitive to low Pi-elicited primary Root Shortening 1), AtHHO2 (HRS1 Homologue 2), and AtHHO3. These data, together with gene co-expression network analysis in Arabidopsis and grape, suggest that the tolerance to cold is in part achieved through the increased synthesis of raffinose family oligosaccharides (RFOs) and that this pathway is possibly controlled by AQ. This study also suggests that AQ may share the role of AtHRS1 under non-cold-stressed conditions (maintaining root phosphate homeostasis), and that as a cold-related gene, it may represent an important candidate gene for molecular breeding of cold-tolerant plants.

## Materials and methods

### Cold treatment of grapevine and Amur grape plantlets

Micropropagated *V. amurensis* and *V. vinifera* cv. ‘Muscat Hamburg’ plantlets were grown on half-strength Murashige and Skoog (1/2 MS; pH 5.8, 30 g l^−1^ sucrose, 0.7% agar) at 26 °C and a 16 h light photoperiod. Six-week-old plantlets were placed in an illuminated incubation chamber at 4 °C. Shoot apices with one fully developed leaf were harvested at 0, 1, 2, 4, 8, 12, 24, and 48 h after cold treatment. Each sample consisted of three plantlets, and three biological replicates were employed.

### Cloning and sequence analysis

The *AQ* ORF was amplified from Amur grape and grapevine leaf cDNAs, with primers AQ-ORF-F and AQ-ORF-R (see Supplementary Table S1 at *JXB* online), designed from the sequence of gene model *VIT_01s0011g03110* in the *V. vinifera* cv. ‘Pinot Noir’ (PN40024) 12Xv1 genome accession (Jaillon *et al.*, 2007). Sequence alignment of AQ with its homologues from Arabidopsis was conducted in DNAMAN 7.0. Phylogenetic trees were constructed using MEGA7.0 software by the maximum likelihood tree method and computed using the WAG model, with g-distributed (G+I) rates among sites and partial deletion gap treatment. Tree nodes were evaluated by bootstrap analysis for 1000 replicates. Nuclear localization signals in the protein sequence of AQ were searched using NLS-mapper (http://nls-mapper.iab.keio.ac.jp/cgi-bin/NLS_Mapper_form.cgi), LocSigDB (http://genome.unmc.edu/LocSigDB/), and NLSdb (https://rostlab.org/services/nlsdb/).

### Gene expression analysis

RNA extraction and quantitative real-time PCR (qRT-PCR) were carried out as described by Sun *et al.* (2015), using three technical and three biological replicates. Primers AQ-RT-F and AQ-RT-R were designed in an identical region between both grape species. Primers are listed in Supplementary Table S1.

### Subcellular localization and gene expression analyses

The ORF of *VaAQ* (excluding its termination codon) was fused to the green fluorescent (GFP) gene downstream of the *Cauliflower mosaic virus* (CaMV) *35S* promoter in the pGFP2 vector. The resulting *35S:VaAQ-GFP* and the control *35S:GFP* constructs were genetically introduced into Arabidopsis mesophyll protoplasts using a polyethylene glycol (PEG) transfection protocol (Yoo *et al.*, 2007). DAPI was used for nuclear staining. Subcellular localization was observed using a Leica TCS SP8 confocal laser-scanning microscope.

### Transformation of Arabidopsis and generation of transgenic plants

The full-length ORF of *VaAQ* was ligated into pCAMBIA1301s driven by the CaMV *35S* promoter. *Agrobacterium* strain GV3101 harbouring the *VaAQ* construct was used to transform the wild-type (WT, Col-0) Arabidopsis plants by the floral dip method (Clough and Bent, 1998). The transformed plants were selected with hygromycin (50 mg l^−1^). Three T_3_ single-insertion homozygous transgenic lines (#1, #2, and #3) were selected for molecular and phenotypic analyses. Seeds of the WT and overexpressing Arabidopsis lines were sown on soil and grown in a greenhouse under standard controlled environmental conditions.

### Cold and freezing treatments in Arabidopsis, electrolyte leakage assay, and physiological analyses of the stress responses

Three-week-old WT and transgenic Arabidopsis plants were subjected to subsequent cold and freezing temperatures. Plants were placed in a growth chamber set at –1 °C for 8 h in the dark. The chamber was then cooled at a rate of –1 °C h^−1^ until –11 °C was reached. After exposure to –11 °C for 3 h, plants were kept at 4 °C for 12 h in the dark and then transferred to standard growth conditions. The survival rate was calculated 3 d later after the end of the entire treatment. Each sample consisted of 14 seedlings, and each experiment was performed in triplicate.

The electrolyte leakage assay was carried out as described in Sun *et al.* (2016) with modifications. Briefly, 3-week-old seedlings of WT and transgenic Arabidopsis plants were acclimated at 4 °C for 3 d. Three rosette leaves were detached, transferred to 10 ml tubes, and placed in a low-temperature illuminated incubation chamber at 0 °C for 1 h. The temperature was subsequently reduced at a rate of –2 °C h^−1^. Tubes were removed at −6, −8, −10, and −12 °C, leaves were thawed overnight at 4 °C, and relative electrical conductivity was measured as previously described. Assays were done in triplicate.

A third analysis consisted of treating 3-week-old WT and transgenic Arabidopsis plants at 4 °C for 3 d. Superoxide dismutase (SOD), peroxidase (POD), and catalase (CAT) activities, along with malondialdehyde (MDA) contents were measured according to the protocol described by Shin *et al.* (2012) and compared with a control (non-treated) sample. Each transgenic line was independently sampled and each sample consisted of a pool of three seedlings (experiments performed in triplicate). For the qRT-PCR analyses, a pool of the three transgenic lines were considered as the experimental unit, with three biological replicates corresponding to three different pools of plants.

### RNA-Seq and bioinformatic analyses of cold responses in WT and transgenic Arabidopsis plants

RNA-Seq analysis was performed using rosette leaves collected from 3-week-old WT and transgenic Arabidopsis plants grown under no stress and cold stress (treated at 4 °C for 3 d) conditions. An equal amount of rosette leaves from three independent transgenic lines (#1, #2, and #3) was pooled and considered as the overexpression line for RNA isolation. Four types of samples were collected, namely WT non-stressed (WT-NS), WT cold-stressed (WT-S), 35S:AQ non-stressed (35S:AQ-NS), and 35S:AQ cold-stressed (35S:AQ-S), sampled with two biological replicates. Single-ended sequencing was performed on a BGISEQ-500 by the Beijing Genomic Institution (BGI, Shenzhen, China).

RNA-Seq data analysis was performed on the AIR platform (www.transcriptomics.cloud). The quality of the raw reads was checked with FastQC (http://www.bioinformatics.babraham.ac.uk/projects/fastqc/), and trimmed to remove low quality bases and sequencing adaptors with the tool BBDuk (https://sourceforge.net/projects/bbmap/). A minimum Phred-like score of 25 was set and a minimum length of the reads of 25 nucleotides. High quality reads were then mapped on the reference genome (TAIR10) of Arabidopsis with STAR (https://github.com/alexdobin/STAR). Read summarization was then performed with featureCounts (http://bioinf.wehi.edu.au/featureCounts/) using only the reads with a mapping quality >30. The statistical analysis was performed with R. Specifically, genes expressed at a low level were removed with the package HTSFilter (http://www.bioconductor.org/packages/release/bioc/html/HTSFilter.html), selecting ‘TMM’ as the normalization method. Then, the filtered genes were used to perform a differential analysis with EdgeR. Genes were considered statistically differentially expressed if the corrected *P*-value (false discovery rate, FDR) was <0.05. Gene Ontology Enrichment Analysis (GOEA) was performed with in house scripts based on a hypergeometric test on the proportion of GO categories between the differentially expressed genes (DEGs) and the whole genome; GO categories were considered enriched if the FDR of the test was <0.05.

The original sequence data were submitted to the database of the NCBI Gene Expression Omnibus under the accession number GSE103964. An expression heatmap was constructed for a pre-selection of genes of interest using the web-based tool Heatmapper (http://www1.heatmapper.ca/expression/), transforming RPKM (reads per kilobase of transcript per million mapped reads) values using a *Z*-score scaling.

### Transformation of *V. amurensis* petiole and generation of transgenic calli

The full-length ORF of *VaAQ* was ligated into the pSAK277 vector under the control of the *35S* promoter and transformed into *V. amurensis* petiole according to Zhao *et al.* (2017). *VaAQ* transgenic calli were confirmed by PCR and RT-PCR. Eight transgenic calli lines were confirmed by detecting *VaAQ* and *NPTII* with primers AQ-ORF-F/AQ-ORF-R, and nptII-F/nptII-R, respectively (Supplementary Table S1). Three *35S:VaAQ* highly expressing T_1_ transgenic calli lines (L1, L2, and L3) were selected for further physiological and metabolic analyses.

### Cold tolerance evaluation of *VaAQ*-overexpressing grapevine calli

A system for differential thermal analysis (DTA) according to Mills *et al.* (2006) was used to assess cold tolerance of grapevine calli. The Keithley Multimeter Data Acquisition System (DAS) (model 2700-DAQ-40) linked with a programmable freezer, the Tenney Environmental Test Chamber (model T2C, Thermal Product Solutions), was used to measure and collect voltage output. *VaAQ*-overexpressing grapevine calli (L1, L2, and L3) were directly placed on the thermoelectric modules (TEMs), and the empty vector (EV) was used as a control. Assays were performed in triplicate. The freezer was programmed to drop to 4 °C in 30 min, hold at 4 °C for 1 h, drop to –16 °C in 10 h (a cooling rate of 2 °C h^−1^), hold at –16 °C for 1 h, then return to 4 °C in 45 min. The DAS recorded signals from each TEM at 15 s intervals. Exotherms were identified manually from a plot of thermistor output (*x*-axis) versus loaded-TEM output minus empty-TEM output (*y*-axis). The low temperature exotherms (LTEs) were used to evaluate the cold tolerance.

### *AQ* gene co-expression network analyses in *V. vinifera*

Our previously established pipeline (Loyola *et al.*, 2016; Wong *et al.*, 2017) was adopted for large-scale processing of publicly available grapevine RNA-Seq data sets (obtained from the NCBI Sequence Read Archive, http://www.ncbi.nlm.nih.gov/sra), with the only difference that the estimation of RNA-Seq-derived transcript abundance was performed with the RPKM method. In this study, an updated RNA-Seq gene expression matrix of 29970 genes by 235 conditions (derived by averaging respective biological replicates from 654 samples; Supplementary Table S2) was used.

Log-transformed RPKM values were used as input for the construction of a gene co-expression network (GCN) based on the mutual rank (MR) metric (Obayashi and Kinoshita, 2009). Using *VvAQ* as the ‘guide’ gene, the top 300 (unless otherwise stated) co-expressed genes (CEGs; ranked by ascending MR value) were extracted and tested for over-represented MapMan BIN functional categories (Lohse *et al.*, 2014) using the Fisher’s exact test adjusted FDR. Incremental enrichment analysis of GO categories was performed with gProfileR using the ‘Ordered query’ option for comparisons (http://biit.cs.ut.ee/gprofiler/index.cgi). An FDR threshold <0.05 was used to determine significantly enriched categories for both ontology schemes. Both network construction and functional enrichment were performed according to previously established workflows (Wong *et al.*, 2017). All networks were visualized with Cytoscape v3.5 (Shannon *et al.*, 2003). Putative orthology between Arabidopsis and grape genes present in the network was tested by Biosequence analysis using profile hidden Markov models (HMMER, http://www.ebi.ac.uk/Tools/hmmer/search/phmmer).

### *Cis*-regulatory element (CRE) enrichment analyses in Arabidopsis and grapevine

Fraction of reads in peaks (FRiP) data for DNA affinity purification-sequencing (DAP-seq) experiments concerning Arabidopsis homologues of VaAQ, namely AtHRS1, AtHHO2, and AtHHO3, were obtained from O’Malley *et al.* (2016). FRiP motif peaks identified/present in the promoter region (1.5 kb upstream of transcription start site, TSS) of Arabidopsis genes that are ≥5% were used to infer target genes of the respective Arabidopsis homologues. Diverse CREs between 6-mer and 8-mer (215 in total) in length were tested for over-representation based on the hypergeometric distribution as previously described (Wong *et al.*, 2017) using Arabidopsis promoter sequences (1.5 kb upstream of TSS) as background. Two analyses were performed: enrichment of CREs in (i) DAP-seq target gene groups using the top 350 hits (descending signal intensity) and (ii) on DEGs in VaAQ-AtOE groups. FDR thresholds of <0.01 (strict) and <0.05 were used to determine significantly enriched CREs. *In silico* CRE analysis in grapevine promoters was conducted with selected CREs in the promoters of *VvAQ*-CEGs as previously described (Wong *et al.*, 2017).

### Quantification of RFOs

Sucrose, raffinose, and stachyose were extracted and quantified through HPLC from WT and transgenic Arabidopsis plants and Amur grape calli, according to Dixit *et al.* (2011).

## Results and Discussion

### Isolation of *AQ* from two grape species with a contrasting cold response

**Fig. 1.**
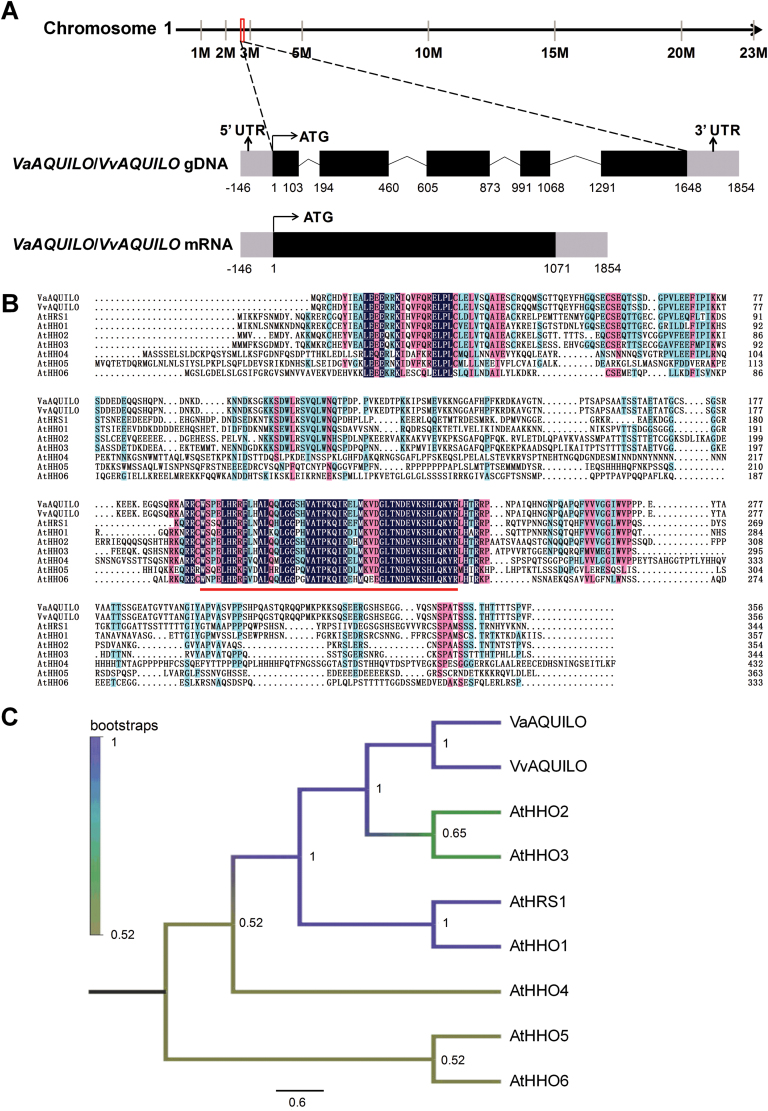
Genomic structure, sequence alignment, and phylogenetic analysis of *AQUILO* (*AQ*) in *Vitis*. (A) Genomic structure of the *AQ* locus. The *AQ* genomic sequence spans 1648 bp and is composed of five exons. The full-length cDNA of *VaAQ*/*VvAQ* contains a 1071 bp ORF. Exons are showed in black and introns as ^. Sequence alignment (B) and phylogenetic tree (C) of grapevine and Amur grape AQ proteins with their closest homologues from Arabidopsis. The conserved GARP domain is shown underlined in red. The protein sequences were aligned using Clustal W2, and the phylogenetic tree was generated with MEGA7.0 using the maximum likelihood tree method with 1000 bootstraps. The branch support values are indicated. The length of the scale bar corresponds to 0.5 substitutions per site. Gene IDs from TAIR are as follows: *AtHRS1* (At1g13300), *AtHHO1* (At3g25790), *AtHHO2* (At1g68670), *AtHHO3* (At1g25550), *AtHHO4* (At2g03500), *AtHHO5* (At4g37180), and *AtHHO6* (At1g49560).

In a previous study we found that the gene *VIT_01s0011g03110* was significantly up-regulated under cold stress (Xin *et al.*, 2013). We isolated this gene from *V. amurensis* and *V. vinifera* cv. ‘Muscat Hamburg’ and designated them as *VaAQUILO* (*VaAQ*; GenBank accession no. MG711316) and *VvAQUILO* (*VvAQ*; GenBank accession no. MG711317), respectively. In both species, the gene length is 1071 bp, encoding a putative protein of 356 amino acids. The corresponding length of both genes is 1648 bp (including five exons), which is mapped to chromosome 1 of the cv. Pinot Noir genome (PN40024) (Fig. 1A). Alignment of VaAQ and VvAQ showed that they shared 99.2% identity. Like their closest homologues found in Arabidopsis, they share the conserved GARP (GOLDEN2, ARR-B, Psr1) domain, found in all GARP/MYB-related transcription factor family members (Rossini *et al.*, 2001; Fig. 1B). A phylogenetic tree based on the full-length protein sequences suggests that AQ shares the closest homology with AtHRS1, AtHHO2, and AtHHO3 (Fig. 1C), implying that AQ proteins may at least share the control of root traits, regulate genes governing phosphate homeostasis, and influence seed germination through abscisic acid (ABA) signalling like their homologues in Arabidopsis (Liu *et al.*, 2009; Wu *et al.*, 2012; Nagarajan *et al.*, 2016).

### Low temperature induces the expression of *AQ* in *Vitis*

Due to their roles in controlling root architecture in response to nutrient sensing, AtHRS1 and AtHHO2 share high expression in roots (Medici *et al.*, 2015; Nagarajan *et al.*, 2016). We searched public transcriptomic data sets for the expression domains of *VvAQ* in grapevine organs and, despite its generally ubiquitous expression, the highest expression was found in roots, vegetative tissues (mature stems or some leaf samples), seedlings, and pollen (Supplementary Fig. S1). Throughout berry development, despite the fact that we see a general decrease from the pre-veraison to ripening stages, in the late ripening progression phase we see a subtle increase (Supplementary Fig. S1A). This response is much more evident in the cv. ‘Corvina’ expression atlas (Supplementary Fig. S1B), where *AQ* is up-regulated by post-harvest withering, a process inducing a strong dehydration stress response in the berry (Zamboni *et al.*, 2008). The expression of these genes was compared with that of *AQ* Arabidopsis homologues by inspecting RNA-Seq gene expression atlases in Genevestigator (Hruz *et al.*, 2008; https://genevestigator.com/). As seen in Supplementary Fig. S2, *AtHRS1* and *AtHHO3* have their highest expression in roots, whereas *HHO2* is mostly expressed in hypocotyl, blades (lamina), and roots. *HHO3* is also widely expressed (though more variably) in shoots and leaves.

**Fig. 2.**
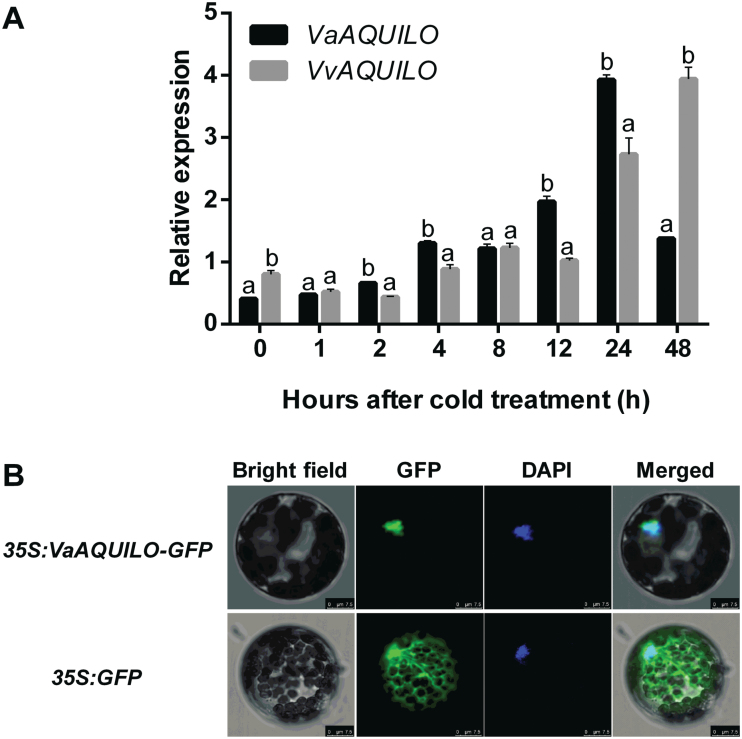
Cold induction and subcellular localization of AQUILO (AQ) in grapevine. (A) Expression pattern of *VaAQ* and *VvAQ* under cold stress in shoot apices and young leaves. *MALATE DEHYDROGENASE* (*MDH*, *VIT_07s0005g03350*) and *ACTIN* (*VIT_04s0044g00580*) genes were used as calibration qRT-PCR controls. Each reaction was performed in triplicate (technical replicates) with three independent biological replicates (*n*=3). The relative expression was calculated using the 2^−ΔΔCt^ method. Error bars represent the SE. Different letters indicate significant differences at a given time point (*P*<0.05; one-way ANOVA). (B) Subcellular localization of VaAQ in Arabidopsis protoplasts. The fluorescent signal generated by a *35S:VaAQ*-GFP construct transfected in Arabidopsis protoplasts was detected by confocal laser scanning microscopy. The images are representative of three independent experiments resulting in the same fluorescence pattern. Scale bars correspond to 10 µm.

As we previously identified *AQ* to be highly induced by cold (Xin *et al.*, 2013), we tested the temporal cold induction pattern of *AQ* in *V. amurensis* and cv. ‘Muscat Hamburg’, two *Vitis* species with contrasting tolerance to cold (Fennell, 2004). We performed qRT-PCR in cold-treated (4 °C) shoot apex samples, which included one well-developed leaf. The expression of *VaAQ* and *VvAQ* rapidly increased after initiating cold stress and peaked at 24 h (9.7-fold) and 48 h (4.9-fold), respectively (Fig. 2A). One-way ANOVA showed that all time points were significantly different between genotypes except at 2 h and 8 h, with a higher expression of *VaAQ* at 2, 4, 12, and 24 h (Fig. 2A). Two-way ANOVA indicated there was a significant ‘genotype×time’ interaction (Supplementary Table S3). These results indicate that *AQ* is induced by cold stress and that this response is earlier in *V. amurensis* than in *V. vinifera* cv. ‘Muscat Hamburg’.

### VaAQ is localized to the nucleus

Sequence analysis tools such as NLS-mapper, LocSigdb, and NLSdb suggested that VaAQ did not contain a nuclear localization signal, but as a hypothetical transcription factor, it should be localized to the nucleus, as seen for its Arabidopsis orthologues AtHRS1 and AtHHO2 (Medici *et al.*, 2015; Nagarajan *et al.*, 2016). We fused the ORF of *VaAQ* to the N-terminus of the GFP protein under the control of the CaMV *35S* promoter. 35S:VaAQ–GFP and a control GFP construct were independently transformed in Arabidopsis protoplast cells. VaAQ–GFP green fluorescence was exclusively detected in the nucleus while GFP signal was evenly distributed in both the cytoplasm and nucleus (Fig. 2B), suggesting that VaAQ exerts its roles in the nucleus, probably affecting transcription.

### Ectopic expression of *VaAQ* enhances cold tolerance in transgenic Arabidopsis

**Fig. 3.**
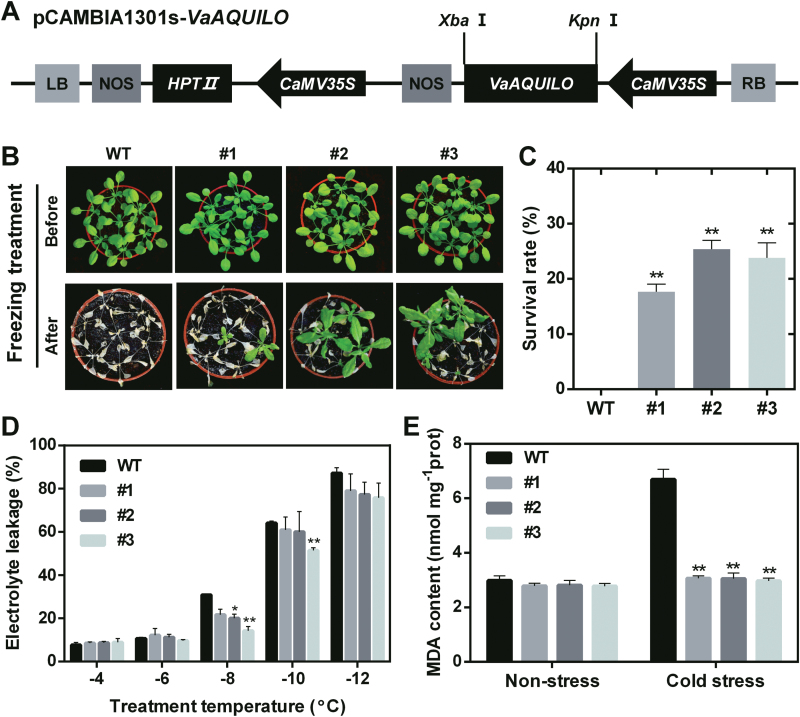
*VaAQUILO* (*VaAQ*) overexpression generates a cold tolerance response in transgenic 3-week-old Arabidopsis plants. (A) Organization of the vector used for transformation of Arabidopsis plants. (B) Phenotypes of the overexpression (OE) lines (#1, #2, and #3) and WT control after cold and freezing treatment. (C) Survival rate of transgenic Arabidopsis after freezing treatment. (D) Electrolyte leakage of OE lines and WT plants treated at –4 °C to –12 °C. (E) MDA contents under non-stress and cold stress (4 °C for 3 d) conditions. Data are mean values ±SE of three biological replicates. Asterisks (**) and (*) indicate significant differences compared with the WT at *P*<0.01 and *P*<0.05 (Student’s t-test), respectively.

To investigate further the function of *VaAQ* in response to cold stress, seven T_3_ homozygous transgenic Arabidopsis lines were generated and three single insertion transgenic lines (#1, #2, and #3) displaying high *VaAQ* expression levels were used in subsequent experiments. To evaluate the function of VaAQ in cold tolerance, 3-week-old WT and transgenic Arabidopsis plants were subjected to a cold and freezing treatment. The results showed that *VaAQ*-overexpressing plants displayed significantly improved cold tolerance (Fig. 3A-B). The average survival rate of the *VaAQ*-overexpressing plants was >20% after freezing stress at −11 °C, whereas all WT plants were frozen to death (Fig. 3C). The electrolyte leakage and MDA content are widely used as indicators for membrane damage, while the latter also indirectly indicates ROS damage as MDA measures a by-product of ROS accumulation in the form of lipid peroxidation products. In this study, the electrolyte leakage of *VaAQ*-overexpressing plants was significantly lower in two of the three Arabidopsis transgenic lines treated at –8°C (Fig. 3D). There was no significant difference in MDA content among transgenic lines and the WT control under non-stress conditions, while in the transgenic plants the content was significantly lower under cold stress (Fig. 3E). These results indicate that VaAQ can reduce membrane damage and that this may represent one of the mechanisms by which AQ improves tolerance to cold stress.

### *VaAQ* overexpression increases antioxidant enzyme activities and up-regulates ROS scavenging-related genes

**Fig. 4.**
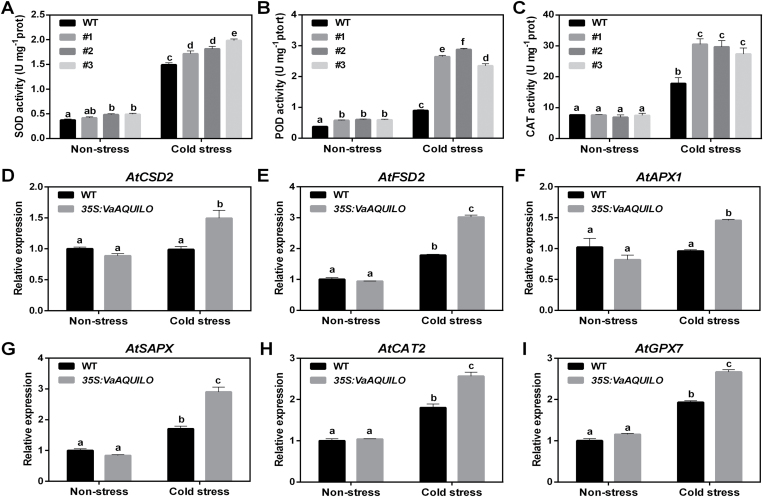
Effect of *VaAQUILO* (*VaAQ*) overexpression (OE) on antioxidant enzyme activities and the expression of ROS-scavenging-related genes. Superoxide dismutase (SOD; A), peroxidase (POD; B), and catalase (CAT; C) activities in OE lines and WT plants under non-stress and cold stress conditions. Gene expression levels of *AtFSD2* (At5g51100; D), *AtCSD2* (At2g28190; E), *AtCAT2* (At4g35090; F), *AtAPX1* (At1g07890; G), *AtSAPX* (At4g08390; H), and *AtGPX7* (At4g31870; I) were monitored under the same conditions. Transcript levels were analysed by qRT-PCR using *ACTIN2* (*ACT2*, AT3G18780) and *UBIQUITIN10* (*UBQ10*, At4g05320) as calibration controls. Gene expression was normalized to the expression obtained in the WT under non-stressed conditions. Each reaction was performed in triplicate (technical replicates). Data are mean values ±SE of three biological replicates (*n*=3). Different letters indicate significant differences compared with the WT under non-stress conditions (*P*<0.05, as determined by one-way ANOVA with a post-hoc LSD test).

To investigate further the physiological changes in *VaAQ*-overexpressing Arabidopsis, we tested the activities of three antioxidant enzymes, namely SOD, POD, and CAT. Previous studies have shown that cold tolerance is indeed affected by these activities *in planta* (Lee and Lee, 2000; Kuk *et al.*, 2003; Zhou *et al.*, 2012). Under non-stressed conditions, overexpression of *VaAQ* had no significant effects on the activities of SOD and CAT (Fig. 4A, C). However, when exposed to 4 °C (stress condition), SOD and CAT displayed significantly higher activities in *VaAQ*-overexpressing plants when compared with WT plants (Fig. 4A, C). In contrast, under both non-stressed and cold-stressed conditions, *VaAQ*-overexpressing plants displayed significantly higher POD activity (Fig. 4B), potentially conferring a more effective antioxidant defence system compared with WT plants (Fig. 4B).

To gain further insights into the molecular mechanism underlying the enhanced antioxidant enzyme activities in *VaAQ*-overexpressing Arabidopsis, the expression patterns of six ROS-scavenging-related genes (Mittler *et al.*, 2004) was examined in WT and transgenic plants under non-stressed and cold stress conditions (Fig. 4D–I). These genes comprised two SOD genes (*AtFSD2* and *AtCSD2*), two ascorbate POD genes (*AtAPX1* and *AtSAPX*), a CAT gene (*AtCAT2*), and a glutathione POD gene (*AtGPX7*). Under non-stressed conditions, there was no significant difference in the expression of these genes between the WT and transgenic plants (Fig. 4D–I). After cold treatment, however, transcript levels of all analysed genes were slightly up-regulated in the *VaAQ*-overexpressing plants and displayed a significantly higher expression in the transgenic versus WT plants (Fig. 4D–I). These results suggest that the increased antioxidant enzyme activities and transcript levels of ROS-scavenging-related genes may play positive roles in improving cold tolerance of *VaAQO*-overexpressing Arabidopsis plants.

### Modulation of cold-responsive gene expression in *VaAQ*-overexpressing Arabidopsis

**Fig. 5.**
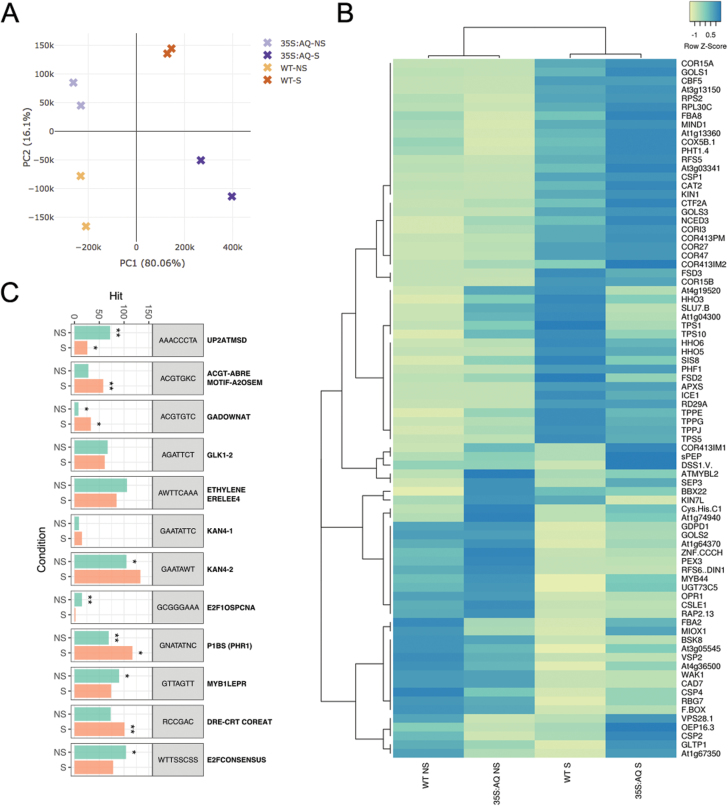
RNA-Seq analysis of *VaAQUILO* (*VaAQ*) overexpression in cold-treated Arabidopsis plants. (A) Principal component analysis (PCA) of WT and *35S:AQ* stressed (cold-treated, S) and non-stressed (NS) data sets, confirming different expression signatures for each condition. The percentage of variation is depicted on the PC1 and PC2 axes. (B) Expression heatmap for a selected list of significantly affected genes (FDR <0.05) related to cold responses, trehalose phosphate and galactinol/raffinose pathways, antioxidant enzymes, phosphate homeostasis, and other highly up-regulated genes in response to *VaAQ* overexpression. Gene IDs, together with their RPKM and log2FC values can be found in Supplementary Table S5. Negative and positive *Z*-scored RPKM values represent low and highly expressed genes, respectively, while values around zero correspond to mild expression. (C) Distribution and enrichment of selected *cis*-regulatory elements (CREs) in the promoter region of *AQ*-up-regulated genes in S and NS conditions. The number of target promoters (Hit) containing the relevant CRE is depicted. Statistically significant CRE observations are represented with asterisks *(0.01>FDR>1.0E-3), **(1.0E-3>FDR>1.0E-5), ***(FDR<1.0E-17). A complete list of CREs in both *35S:AQ* data sets is found in Supplementary Table S7.

Since Arabidopsis plants overexpressing *VaAQ* had increased tolerance to cold and higher antioxidant enzyme activities than WT plants, we hypothesized that an increased activation of the cold response pathway was directly exerted through VaAQ. mRNA libraries were constructed for the four previously tested samples (WT and 35S:VaAQ in cold and non-stressed conditions) and sequenced by BGISEQ-500. After adaptor and low-quality base trimming, 185422178 clean reads (23.2 million reads in average per condition) remained. An average of 94.1% of reads mapped uniquely to the reference genome, while 5.38% of reads mapped to multiple loci and were discarded. Principal component analysis (PCA) showed that a majority of the variation in abundances of mRNAs between libraries is associated with cold stress (PC1 of 80%; Fig. 5A), while PC2 was inferred to capture predominantly transgene-induced variation (16%). The differential expression analysis, run through edgeR, showed 1940 and 2205 genes to be up- and down-regulated (FDR <0.05) by *VaAQ* overexpression in the stressed condition, compared with 345 and 233 in the non-stressed condition (Supplementary Table S4). This indicates that the biggest differences between the WT and 35S:VaAQ, in terms of the number of DEGs, are found when the cold stress is imposed.

We further inspected those genes with the highest expression differences between WT and *VaAQ*-overexpressing plants. The expression heatmap in Fig. 5B shows a selection of such genes, including canonical cold pathway marker genes. Hierarchical clustering analysis showed that DEGs were mainly classified into four major categories: (i) genes induced by cold but even more by *VaAQ* (suggesting a synergistic role of *AQ* and the cold pathway); (ii) those exclusively induced by cold; (iii) genes induced by *VaAQ* but repressed by cold itself; and (iv) those repressed by cold and *VaAQ* overexpression. We found a significant up-regulation of a set of cold-related genes (*COR* genes) many of which were more induced in the *35S:VaAQ* condition (Fig. 5B; Supplementary Table S5). Other interesting genes falling into category (i) correspond to genes coding for galactinol synthases (GoLS1/3) and a raffinose synthase (RFS5), both types of enzymes participating in the synthesis of RFOs. These sugars have been previously associated in Arabidopsis with chilling stress tolerance and as plant cell protectants from oxidative damage (Nishizawa *et al.*, 2008; Nishizawa-Yokoi *et al.*, 2008). Two phosphate-related genes were also up-regulated by cold and AQ, namely *INORGANIC PHOSPHATE TRANSPORTER 1-4* (*PHT1-4*) and *PHOSPHATE TRANSPORTER TRAFFIC FACILITATOR 1* (*PHF1*), suggesting that AQ may share the roles of AtHRS1 and AtHHO2 (and potentially AtHHO3) in phosphate homeostasis (Liu *et al.*, 2009; Nagarajan *et al.*, 2016) and also that cold itself may affect phosphate metabolism through AQ’s function. Among the most highly induced genes in all *35S:VaAQ* conditions, we found a short ORF potentially coding for a small peptide (sORF-encoded polypeptide or SEP, At3g29633) with an as yet unknown function, and a cysteine/histidine-rich C1 domain family protein (At3g27473), which could represent interesting direct targets of AQ in Arabidopsis.

Despite the fact that *CBF* genes were not identified as DEGs in our RNA-Seq data, many genes belonging to the CBF pathway were indeed up-regulated. To test the activation of the CBF pathway by cold and/or *VaAQ* overexpression, we analysed the expression of *AtCBF1,2,3*, *AtICE1*, *AtCOR15A*, *AtCOR47*, *AtKIN1*, *AtRD29A*, and *AtGoLS3* by qPCR. Under no cold stress, transcript levels of *CBF* genes in the *VaAQ* overexpressors were significantly higher than those in the WT. However, after cold treatment, these genes were rapidly increased in the WT, not differing from the cold-treated *VaAQ*-overexpressing plants (Supplementary Fig. S3). From all tested genes, the clearest response was for *GoLS3*, whose transcript levels were higher in the transgenic Arabidopsis than in the WT, in both non-stressed and stressed conditions. These results suggest that *AQ* may either participate as part of the CBF pathway or belong to a pathway crosstalking with CBFs to enhance the cold tolerance in Arabidopsis.

We compared the proportion of GO categories between the genes differentially expressed upon *AQ* overexpression and the whole genome, and found that different stress responses were enriched (Supplementary Table S6). Interestingly, among up-regulated genes in the *35S:VaAQ* versus WT cold stress comparison, there is an enrichment of ‘cold acclimation’ (FDR=2.3E-08) and ‘cellular response to cold’ (FDR=0.00013) terms, suggesting that *AQ* overexpression increases the extent of the cold response itself. Other highly enriched biological processes were ‘response to water deprivation’ (FDR=7.7E-16) and ‘response to oxidative stress’ (FDR=2.3E-15). The molecular function ‘galactinol-sucrose galactosyltransferase activity’ category was also found with an FDR=0.01. In the non-stress comparison, *VaAQ* overexpression generates an enrichment of the molecular function term ‘hydrolase activity, acting on acid anhydrides, in phosphorus-containing anhydrides’ (FDR=3.0E-12) and the biological processes ‘chromatin remodelling’ (FDR=9.7E-12), including ‘histone H3-K9 demethylation’ (FDR=5.3E-07) and many other related processes. The terms ‘cellular response to phosphate starvation’ and ‘phosphate ion transport’ were found with FDR values of 0.06 and 0.08, respectively.

To complement the GO enrichment findings, we performed a CRE enrichment analysis in promoter sequences (1.5 kb upstream of the TSS) of up-regulated genes in the Arabidopsis *35S:VaAQ* groups (Supplementary Table S7). As seen in Fig. 5C, the non-stressed comparison data set was highly enriched with KAN4-2, GADOWNAT (germination and GA-related), P1BS (PHR1-binding sequence), MYB1LEPR, and E2F transcription factor-binding motifs (TFBMs), while the stress comparison was over-represented with the ABA drought-related ABRE and DRE motifs, GADOWNAT, and P1BS, among many others (Supplementary Table S7). KAN4 is recognized by KANADI4/ABERRANT TESTA SHAPE (At5g42630; Merelo *et al.*, 2013), which belongs, like AtHRS1 and AQ, to the GARP family of transcription factors. P1BS instead is bound by PHOSPHATE STARVATION RESPONSE 1 (PHR1; Rubio *et al.*, 2001). The promoter of HHO2 is in fact enriched with P1BS motifs and it was demonstrated that PHR1 exerts a regulatory effect on HHO2 (Nagarajan *et al.*, 2016). These results add more lines of evidence to the idea that AQ increases the response to stress but that in non-stress conditions it may regulate other processes including phosphate homeostasis.

Recently, O’Malley *et al.* (2016) defined the promoter binding preferences for 529 transcription factors in Arabidopsis by using a DAP-Seq technique. Thus, we decided to compare the distribution of CRE from our RNA-Seq lists with those in the promoter regions of AtHRS1, AtHHO2, and AtHHO3 high confidence target genes. For this, we re-analysed the DAP-Seq data, by identifying peaks (FRiP ≥5%) that were only present in the promoter region (1.5 kb upstream of the TSS) of Arabidopsis genes (Supplementary Table S8). This analysis revealed that 328, 3441, and 4025 genes were potential targets of AtHRS1, AtHHO2, and AtHHO3, respectively. CRE analysis using high confidence (top ~350) bound genes resulted in the strong enrichments of GLK1, and P1BS-binding sites and especially KAN4 (Supplementary Fig. S4). It is worth mentioning that many of the genes from Fig. 5B correspond to AtHRS1, AtHHO2, and AtHHO3 targets based on this DAP-Seq re-analysis (Supplementary Tables S5, S8).

### Overexpression of *VaAQ* improves cold tolerance of Amur grape

**Fig. 6.**
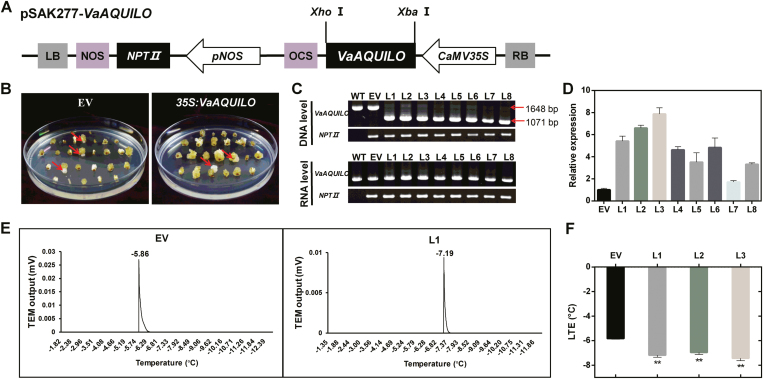
Overexpression of *VaAQUILO* (*VaAQ*) enhances cold tolerance in transgenic Amur grape calli. (A) Vector construction for transformation of *V. amurensis*. (B) Transgenic grapevine calli introduced with empty vector (EV) and pSAK277-*VaAQ*. Positive transgenic calli are indicated with a red arrow. (C) Detection of *VaAQ* and *NPTII* genes at the DNA and mRNA level in the transgenic grapevine calli. Eight transgenic lines (L1–L8) were obtained and verified for DNA insertion and transcript expression of *VaAQ*. (D) *VaAQ* expression in transgenic grapevine calli. (E) Cold tolerance evaluation system of grapevine calli. After placing the calli on the thermoelectric modules (TEMs), exotherms were identified manually from a plot of thermistor output (*x*-axis) versus loaded TEM output minus the empty TEM output (*y*-axis). The low temperature exotherms (LTEs, indicating the cell freeze temperatures) were obtained to evaluate the cold tolerance. (F) LTEs of transgenic grapevine calli. Data are mean values ±SE of three biological replicates. Asterisks (**) and (*) indicate significant differences compared with the EV at *P*<0.01 and *P*<0.05 (Student’s *t*-test), respectively.

We studied the potential role of *VaAQ* in grape by transforming *V. amurensis* petiole explants. After calli were initiated and selected by kanamycin (Fig. 6A, B), eight transgenic lines were confirmed by detecting *VaAQ* and *NPTII* at the DNA and mRNA level (Fig. 6C). The qRT-PCR analysis showed that the expression of *VaAQ* was up-regulated in these transgenic calli lines, with the biggest fold change (FC) being 7.9-fold and the smallest FC 1.7-fold when compared with calli transformed with an empty vector (Fig. 6D). It is worth noting that the levels of *VaAQ* expression obtained in the three high overexpressing calli lines were similar to the peak FC seen in the cold-treated *V. amurensis* leaves/shoot apices (Fig. 2). Three high expression lines (L1, L2, and L3) were selected for the cold tolerance studies, and the EV was used as control. The cold tolerance of *VaAQ*-overexpressing calli was evaluated by LTEs as described by Mills *et al.* (2006) and Muniz *et al.* (1991). The LTEs of EV, L1, L2, and L3 were –5.8, –7.2, –7.0, and –7.4 °C, respectively (Fig. 6E; Supplementary Fig. S5), supporting the hypothesis of *VaAQ* overexpression enhancing cold tolerance also in *Vitis*.

### Gene network analysis positions AQ as a cold-induced hub controlling the accumulation of the RFOs

Since *AQ* overexpression in grape calli led to cold tolerance as in Arabidopsis, we further tested whether the regulatory networks associated with *AQ* heterologous/homologous expression were conserved between the two species. To test this, we performed a systems-oriented analysis by coupling the identification of tightly interconnected relationships (co-expression) to TFBSs in grapevine, and comparing it with the RNA-Seq data obtained in Arabidopsis. We constructed a condition-independent network from RNA-Seq experiments (listed in Supplementary Table S2), and integrated it with the presence of G2-type CREs in the promoter regions of all CEGs. This would enable the identification of overlapping AQ targets in Arabidopsis and grape, allowing determination of the pathways under which this transcription factor enhances cold tolerance.

GO and MapMan (MM) enrichment analyses conducted using the top 300 AQ-CEGs revealed that terms related to ‘cellular polysaccharide biosynthetic process’ (GO), ‘glycerophospholipid catabolic process’ (GO), ‘phosphate ion homeostasis’ (GO), ‘cell wall biogenesis’ (GO and MM), and ‘phenylpropanoids’ (GO and MM) were highly enriched (Supplementary Table S9). In relation to the terms ‘cell wall’ and ‘cellulose metabolic process’, we found 10 cellulose synthase genes, while for the term ‘minor sugar metabolism-raffinose family’, one GoLS and one raffinose synthase were highly co-expressed with AQ, with at least five other *GoLS* genes being co-expressed but found below the top 300 CEGs. Interestingly, the *AQ* co-expression list was also enriched in hormone-related terms such as ‘abscisic acid metabolism’ (MM) and ‘jasmonate metabolism’ (MM), and *AQ* was also co-expressed with biotic and abiotic stress response genes such as R proteins, chitinases, and *RD22* genes (Supplementary Table S9). Several WRKY, MYB, and ethylene-responsive transcription factors were highly represented.

To test for putative AQ DNA binding preferences, we first search for G2-type binding sites in the DAP-Seq data of O’Malley *et al.* (2016), finding two major consensus sequences which were also conserved for AtHRS1, AtHHO2, and AtHHO3 (Supplementary Fig. S6): AKATTCY and RGAATMT. We screened the promoter regions (1.5 kb upstream of the TSS) of the top 300 grapevine AQ-CEGs for the presence of these TFBSs. We also looked for P1BS (GnATATnC) as this CRE is enriched in all AtHRS1/HHO2/HHO3 target lists (Supplementary Fig. S4) and also in the 35S:AQ stressed and non-stressed Arabidopsis overexpression data sets (Fig. 5C). We identified AKATTCY, RGAATMT, and P1BS box signatures in 66.7% (200 genes), 62.0% (186 genes), and 42.3% (127 genes) of the top 300 CEG promoters, respectively, with all three motifs showing significant enrichment with an FDR of 1.31E-05, 3.92E-05, and 2.09E-02. These motifs showed significantly higher frequencies compared with background frequencies in all grapevine promoters predicted to contain these motifs (also assuming 1.5 kb); these signatures were present in 16378 (~55%), 15113 (~50%), and 11616 (~39%) genes, respectively, from a total of 29970 sequences.

**Fig. 7.**
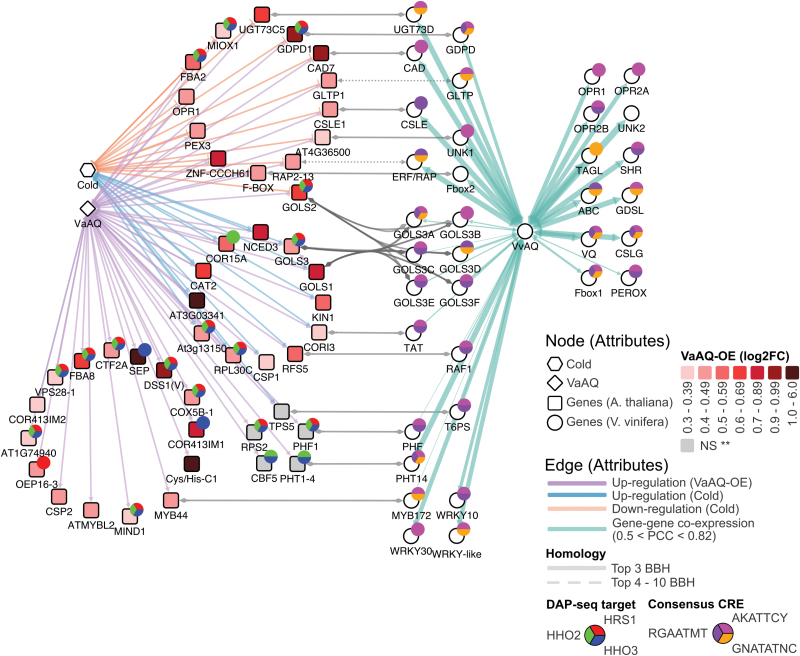
Interspecies regulatory and community gene co-expression network of *AQUILO* (*AQ*). Arabidopsis genes (square nodes) induced by *VaAQ* overexpression and differentially regulated by cold (unless denoted) are shown in the subnetwork in the left panel, while grapevine genes (circle nodes) co-expressed with *VvAQ* in public RNA-Seq data sets are depicted on the right. Arabidopsis nodes are colour-mapped from light to dark red according to the ascending fold change differences (log2FC) identified in the *35S:AQ*-Stress versus WT-Stress RNA-Seq comparison. Grey nodes represent a major up-regulation in the *35S:AQ*-Stress versus WT non-stress comparison. The light orange and blue edges connecting the cold hexagon node and Arabidopsis genes represent significant (FDR <0.05) down-regulation and up-regulation of the latter by the cold treatment. Grey edges connecting Arabidopsis and grapevine genes of the two subnetworks represent high confidence cases of orthology as suggested by biosequence analysis using profile hidden Markov models (pHMMERs). Circle nodes shown as pie charts alongside Arabidopsis genes indicate whether genes are AtHRS1, AtHH2, and/or AtHHO3 direct targets (derived from the re-analysis of DAP-Seq data generated by O’Malley *et al.*, 2016). Pie charts alongside grapevine genes (on the right) represent the presence of G2-type and PBS1 consensus CREs present in the promoter region of the respective co-expressed gene. Edge thickness in the grape co-expression subnetwork represents significant co-expression between *VvAQ* and each gene ranging from 0.5 to 0.82 PCC. Gene IDs can be found in Supplementary Table S8.

Putting together all these results, we constructed an integrated AQ community network for comparing grapevine co-expression data with the RNA-Seq data obtained for the overexpression of AQ in Arabidopsis. This overlapping network shows AQ putative target genes in Arabidopsis and grapevine (Fig. 7). For construction of this network, we included different criteria: (i) grape CEGs with TFBS support; (ii) Arabidopsis genes induced by AQ overexpression in stressed or non-stressed conditions; (iii) Arabidopsis genes induced or repressed by cold; and (iv) Arabidopsis genes being targets of AtHRS1, AtHHO2, and/or AtHHO3 inferred from the re-analysis of Arabidopsis DAP-Seq data (O’Malley *et al.*, 2016). AQ candidate direct targets may represent pathway markers for both phosphate homeostasis and cold-related responses in grapevine. Among these, we suggest *PHT1-4* (*VIT_05s0049g00930*), *PHF* (*VIT_13s0074g00010*), *GLYCEROPHOSPHORYL DIESTER PHOSPHODIESTERASE* (*GDP*, *VIT_14s0068g01500*), and several *GoLS3* (*A–F*) genes as good candidates for further characterization studies.

As seen from our network analysis, AQ has the potential to regulate the galactinol/raffinose pathway in response to cold stress. As this function has not been established before for any of the Arabidopsis HRS1 homologues, we first searched to determine whether the AtHRS1 subfamily (composed of seven members) could be cold responsive, as demonstrated for AQ. Inspection of microarray and RNA-Seq data in Genevestigator allowed us to detect a cold induction pattern for *HHO2*, *HHO3*, and *HHO6* in shoot tissues, while *HRS1* was restricted to roots with no apparent induction under cold treatments (data not shown). Indeed, *HHO3* and *HHO6* were cold induced in our RNA-Seq data (Supplementary Table S5).

Galactinol synthases and raffinose synthases are closely related glycosyl transferases/hydrolases (Supplementary Fig. S7A–C) involved in the biosynthesis of RFOs that function as osmoprotectants, promoting plant stress tolerance in response to heat, chilling, salinity, and methylviologen (Panikulangara *et al.*, 2004; Li *et al.*, 2007; Nishizawa *et al.*, 2008). As seen in Genevestigator, *GoLS1*, *GoLS3*, and *RFS5* are the most cold-induced genes in Arabidopsis (Supplementary Fig. S8). Their closest homologues in grape are highly induced by cold, as seen in an RNA-Seq experiment consisting of 6-week-old micropropagated plantlets (*V. vinifera* and *V. amurensis*) exposed to 4 °C for 0, 2, 4, 8, 24, and 48 h (Supplementary Fig. S7B; H. Xin *et al.*, unpublished).

**Fig. 8.**
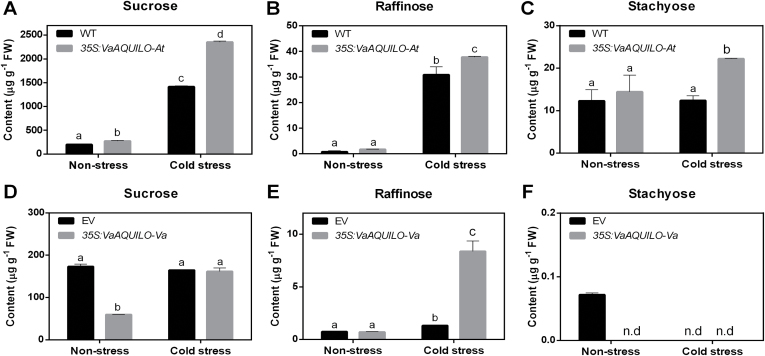
*VaAQUILO* (*VaAQ*) overexpression promotes the accumulation of raffinose family oligosaccharides (RFOs) in *A. thaliana* and *V. amurensis*. Sucrose, raffinose, and stachyose contents of *VaAQ*-overexpressing lines in Arabidopsis (A–C) and Amur grape calli (D–F) under non-stress and cold stress conditions. For Arabidopsis, 3-week-old plants of the wild type (WT) and three transgenic lines (#1, #2, and #3) were subjected to 4 °C for 3 d. For Amur grape calli, 2-week-old calli of empty vector (EV) and three *VaAQ*-overexpressing lines (L1, L2, and L3) were treated at 4 °C for 3 d. Data are mean values ±SE of three biological replicates. Different letters indicate significant differences compared with the WT or EV under non-stress conditions at *P*<0.05, as determined by one-way ANOVA with a post-hoc LSD test.

Due to the *GoLS* and *RSF/RAF* cold induction and their predicted regulation by G2-type transcription factors, we hypothesized that AQ may increase the accumulation of RFOs in cold-treated 35S:AQ Arabidopsis and grapevine plants. To confirm this hypothesis, we tested the accumulation of sucrose, raffinose, and stachyose in transgenic Arabidopsis plants and Amur grape calli. Under non-stressed conditions, raffinose and stachyose levels remained constant after *VaAQ* was overexpressed (Fig. 8). Sucrose was slightly increased in the transgenic Arabidopsis, while it was deceased in the transgenic Amur grape calli (Fig. 8). When the transgenic Arabidopsis plants were exposed to cold stress (4 °C for 3 d), all of the three RFOs were increased significantly compared with the WT plants (Fig. 8A–C). Raffinose was up to 6.3 times higher in transgenic Amur grape calli than in the EV control (Fig. 8E). These results indicate that in response to cold, *VaAQ* increases the accumulation of RFOs in Arabidopsis and Amur grape calli and that this could be one additional mechanism by which AQ increases cold tolerance.

### Final remarks

In this study, we characterized a cold-responsive MYB-like GARP-type transcription factor, AQUILO, from *Vitis*. Its overexpression enhanced the tolerance of Arabidopsis and Amur grapes to low temperatures, suggesting its role as a positive regulator of cold resistance. Its Arabidopsis, orthologues *AtHRS1* and *AtHHO2* have been mainly characterized in phosphate starvation responses, being rapidly induced by phosphate deficiency while it is repressed by nitrate deprivation or other salt stresses (Mito *et al.*, 2011; Medici *et al.*, 2015; Menz *et al.*, 2016). Both genes integrate these responses in the adaptation of root architecture (Medici *et al.*, 2015). As expected, their expression is high in roots. However, *AtHHO2* (and its close homologue *AtHHO3*) are also highly expressed in vegetative tissues (Supplementary Fig. S2), suggesting additional roles for these transcription factors. In agreement with AQ’s identified role in cold responses, and based on gene expression patterns, the set of target genes identified in the re-analysis of Arabidopsis DAP-Seq data (O’Malley *et al.*, 2016), and on the effects of overexpressing AQ in Arabidopsis, we also propose that its closest homologues may also be part of a cold response pathway, a role that has not been asserted before in Arabidopsis.

The increased RFOs in the *AQ*-overexpressing Arabidopsis and Amur grape calli may be caused by increased GoLS activity via transcriptional regulation by *AQ*. The ectopic expression of AQ in Arabidopsis induced *AtGoLS1*, *AtGoLS2*, *AtGoLS3*, and also two raffinose synthase genes (*AtRFS5* and *AtDIN10*) all contributing to the RFO pathway. In cold-treated grapevine and Amur grape apices, we also observed the induction of several gene members of the *GoLS* and *RAF* families, in temporal agreement with the up-regulation of *AQ*. These data correlate to the identification of putative AQ-binding sites in *GoLS* and *RAF* gene promoters, and to the fact that AtHRS1, AtHHO2, and AtHHO3 bind *GoLS* genes. The cold induction of *GoLS* genes has been reported in Arabidopsis and other species (Taji *et al.*, 2002; Shimosaka and Ozawa, 2015), suggesting a conserved mechanism for coping with the detrimental effects of low temperatures on plant growth and development. Accumulation of RFOs has been reported to play a role in protection against drought, high salinity, and many other stresses. From these types of sugars, raffinose was described as the major contributor to cold tolerance in Arabidopsis and petunia (Pennycooke *et al.*, 2003). In agreement with this, our results show that raffinose significantly increases in both WT Arabidopsis and EV Amur grape calli after cold treatment. Meanwhile, the amount of raffinose in 35S:AQ Arabidopsis and Amur grape upon cold treatment significantly increased much more than their controls, suggesting AQ as a key RFO regulator for protection against cold stress in *Vitis* (Supplementary Fig. S9).

The underlying mechanisms explaining how the accumulation of RFOs increases cold tolerance are not completely understood. They have been proposed to protect plant cells from oxidative damage (Van den Ende *et al.*, 2011; Peshev *et al.*, 2013; ElSayed *et al.*, 2014). In this sense, cold stress gives rise to high concentrations of ROS, which are associated with oxidative damage at the cellular level (Blokhina *et al.*, 2003), and RFOs may contribute to overall cellular ROS homeostasis by scavenging processes in the vicinity of organellar membranes (Van den Ende, 2013). *In vitro* experiments with thylakoids during freezing showed the ability of raffinose to protect membranes better than sucrose under stress conditions (Hincha *et al.*, 1990). In addition to RFOs, we also detected the higher activity of major ROS-scavenging mechanisms including SOD, POD, and CAT activities and the expression levels of ROS scavenging-related genes (Mittler, 2002). Previous studies have shown that cold tolerance is affected by these antioxidant activities *in planta* (Lee and Lee, 2000; Kuk *et al.*, 2003; Zhou *et al.*, 2012). Altogether, our results suggest that increased antioxidant enzyme activities and transcript levels of ROS scavenging-related genes, in addition to high raffinose levels, may play a determining role in acquiring cold tolerance via *AQ* up-regulation (Supplementary Fig. S9). One approach to demonstrate this would be to test if AQ knockout dampens the up-regulation of RFO-related genes and whether this prevents accumulation of raffinose in response to cold.

Whether AQ forms part of the CBF pathway is something still to be demonstrated. One certain piece of evidence is that AQ overexpression led to the up-regulation of many genes belonging to the CBF pathway. Like the increased activation of *AtCBF* gene expression in 35S:AQ Arabidopsis plants, we also observed an up-regulation of *VaCBF1–VaCBF4* genes in transgenic grapevine calli after cold treatment at 4 °C for 8 h (Supplementary Fig. S10). In addition, ABA-related CREs were found to be enriched in 35S:AQ-up-regulated genes, while ABA is known to induce the CBF pathway (Knight *et al.*, 2004). These observations suggest that *AQ* may improve the cold tolerance of grapevine via regulating the expression of *CBF* genes, but this does not necessarily mean that AQ is upstream of CBF functions. The presence of CBF-binding sites in AQ and G2-type *cis*-elements in grape *CBF* genes (data not shown) suggests a positive feedback loop where AQ and CBF promote their own expression to initiate cold response cascades rapidly.

## Supplementary data

Supplementary data are available at *JXB* online.

Table S1. List of primers used in this study.

Table S2. Summarized metadata from public RNA-Seq experiments used in the gene co-expression association studies.

Table S3. Two-way ANOVA for the expression of VaAQUILO and VvAQUILO under a cold stress time series.

Table S4. Lists of Arabidopsis differentially expressed genes derived from RNA-Seq experiments.

Table S5. List of differentially expressed genes (FDR <0.05) with high expression differences between WT and *VaAQ*-overexpressing plants, including canonical cold pathway markers, and genes related to trehalose phosphate and galactinol/raffinose pathways, antioxidant enzymes, and phosphate homeostasis.

Table S6. Gene Ontology Enrichment Analysis (GOEA) in RNA-Seq results from 35S:AQ versus WT stressed and non-stressed comparisons.

Table S7. Summary of over-represented (FDR <0.05) *cis*-regulatory elements (CREs) in promoters of significantly up-regulated genes in the Arabidopsis *35S:VaAQ* groups: non-stress and cold stress.

Table S8. List of AtHRS1, AtHHO2, and AtHHO3 targets as inferred from the re-analysis of Arabidopsis DAP-Seq data.

Table S9. Comprehensive list and detailed description of grapevine AQ co-expressed genes (CEGs).

Fig. S1. Gene expression domains of *VvAQUILO* in grapevine organs and throughout development.

Fig. S2. Gene expression anatomy atlases of (A) AtHRS1, (B) AtHHO2, and (C) AtHHO3 retrieved from RNA-Seq public datasets.

Fig. S3. Expression analysis of cold-responsive genes in transgenic Arabidopsis under non-stress and cold stress conditions.

Fig. S4. Distribution and enrichment of selected *cis*-regulatory elements (CREs) in the promoter region of *AtHHO2*, *AtHHO3*, and *AtHRS1* high confidence target genes.

Fig. S5. Profiles of differential thermal analysis (DTA) for low temperature exotherms (LTEs) in Amur grape.

Fig. S6. Transcription factor-binding motifs for the G2-type transcription factors AtHRS1, AtHHO2, and AtHHO3.

Fig. S7. Cold-induced galactinol and raffinose synthases in *Vitis*.

Fig. S8. Cold induction of Arabidopsis *GoLS* and *RFS* genes.

Fig. S9. Schematic representation of AQUILO-related processes identified in this work in relation to the cold response pathway.

Fig. S10. Differential gene expression of *CBF1–CBF4* in the *VaAQ*-overexpressing Amur grape calli.

Supplementary Table S1

Supplementary Table S2

Supplementary Table S3

Supplementary Table S4

Supplementary Table S5

Supplementary Table S6

Supplementary Table S7

Supplementary Table S8

Supplementary Table S9

Supplementary Figures S1-S10
