# Supplementary material for: The GARP/MYB-related grape transcription factor AQUILO improves cold tolerance and promotes the accumulation of raffinose family oligosaccharides
Source: J Exp Bot. 2018 Jan 29;69(7):1749–64. doi: 10.1093/jxb/ery020 (PMC5888914; doi:10.1093/jxb/ery020)

Supplementary Material

Fig. S1. Gene expression domains of *VvAQUILO* in grapevine organs and throughout development. RNA-Seq (A) and cv. Corvina Nimblegen microarray (B) expression data was retrieved from public repositories and normalized. Log2-transformed FPKM (A) and RMA (B) values were then graphed. Probe signals below the 20th percentile are considered as background (~7.5). Colours correspond to type of organ (different green tones: vegetative organs, grey: inflorescence and flower organs, blue: roots, purple: buds, yellow/orange: different sets of berry tissues.

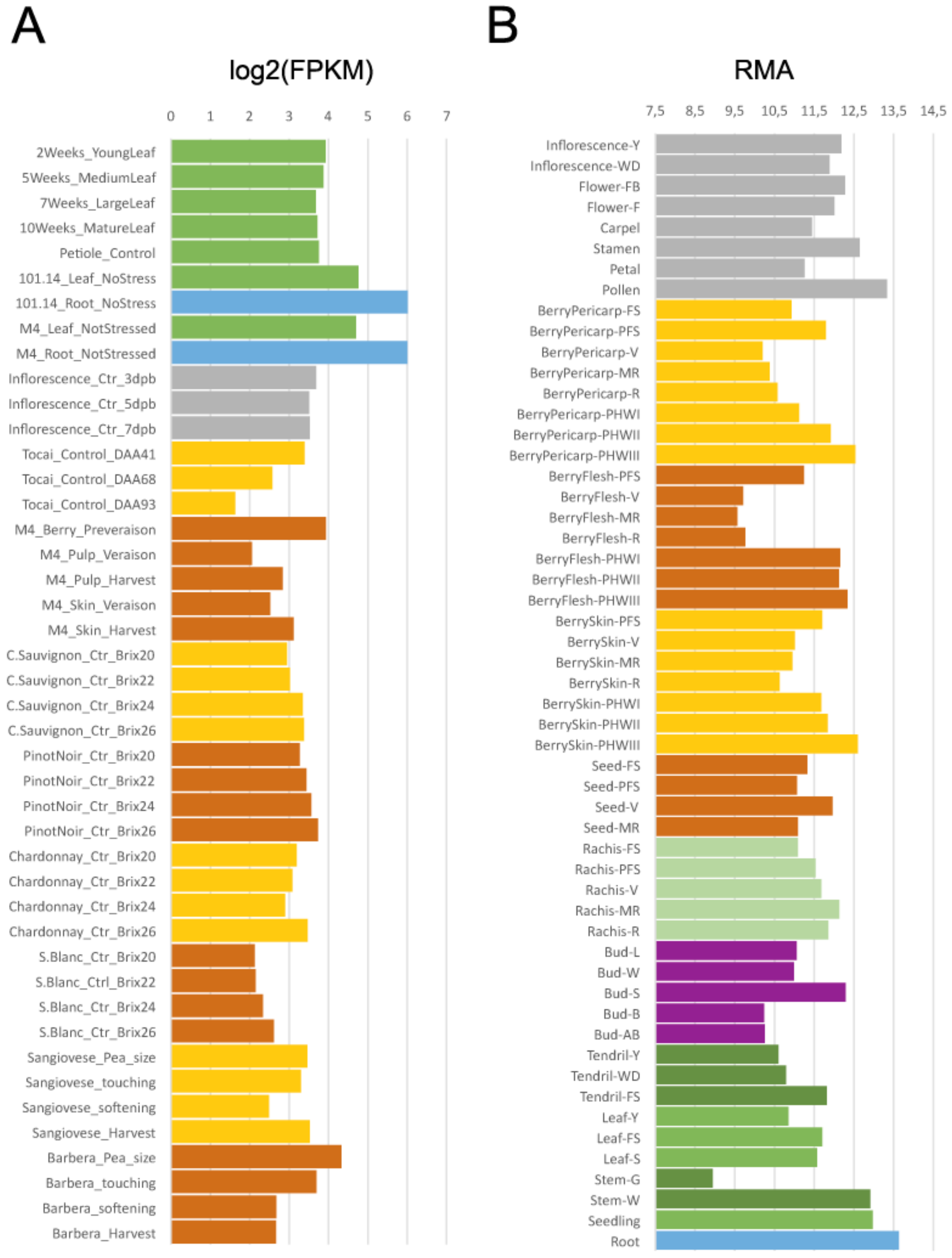

Fig. S2. Gene expression anatomy atlases of (A) AtHRS1, (B) AtHHO2 and (C) AtHHO3 retrieved from RNA-Seq public datasets. 1031 samples were chosen and visualized (log2) in Genevestigator.

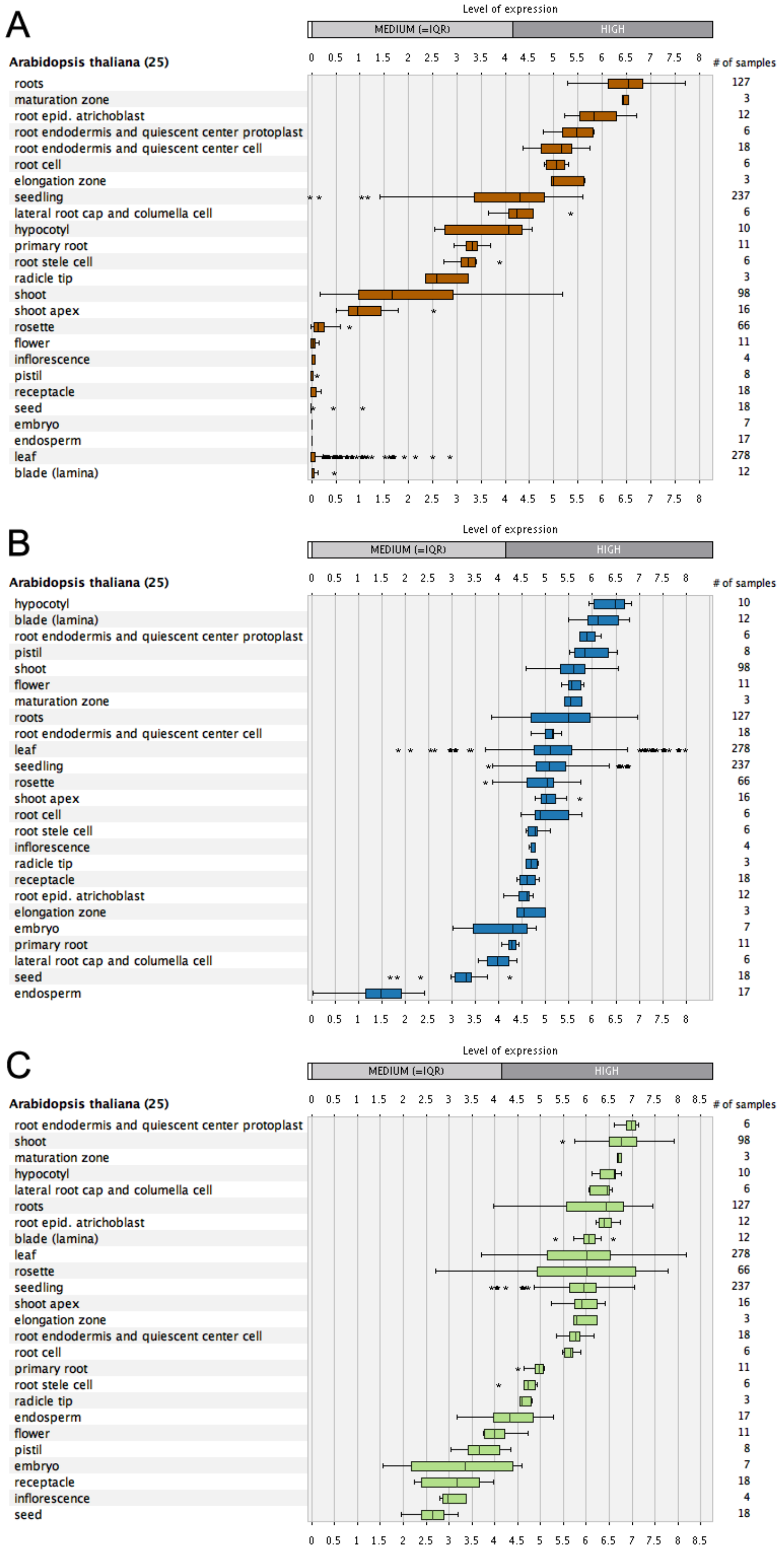

Fig. S3. Expression analysis of cold-responsive genes in transgenic Arabidopsis under non-stress and cold stress conditions. Transcript levels were analyzed by qRT-PCR using *ACT2* (At3g18780) and *UBQ10* (At4g05320) as calibration controls. Gene expression was normalized regarding the expression obtained from WT under non-stress condition. Each reaction was performed in triplicate (technical replicates) with three independent biological replicates (n=3). Data are mean values ± SE of three biological replicates.

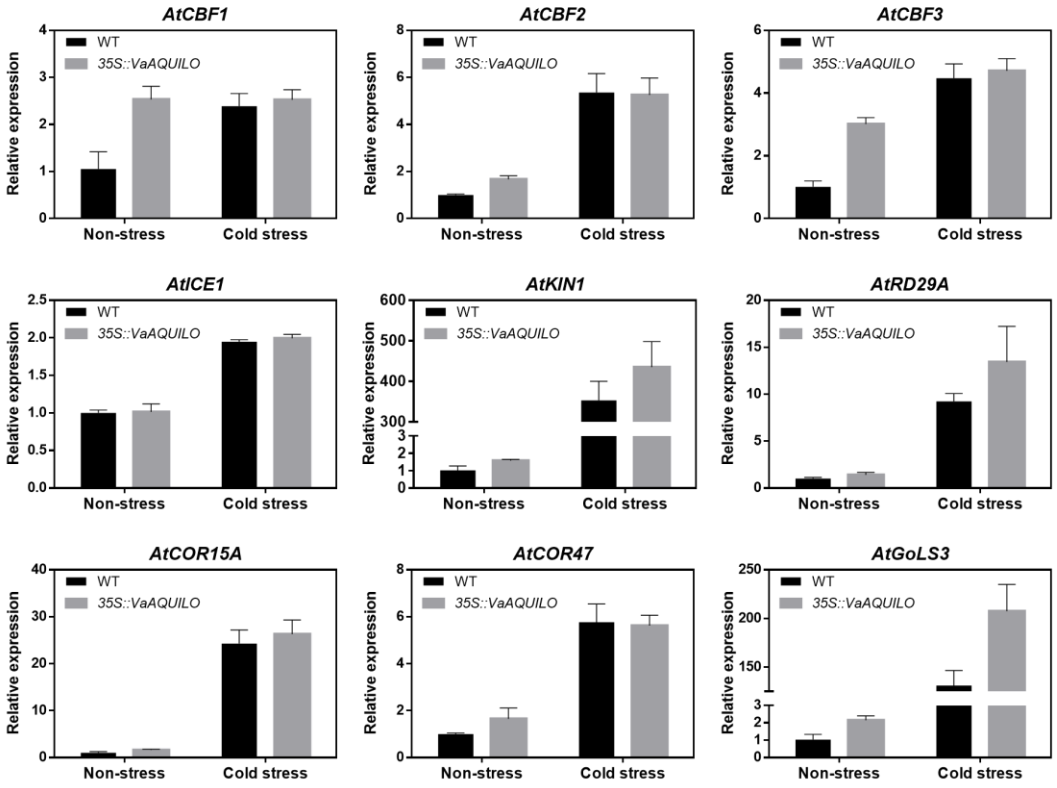

Fig. S4. Distribution and enrichment of selected *cis*-regulatory elements (CRE) in the promoter region of AtHHO2, AtHHO3, and AtHRS1 high-confident target genes. List of putative target genes are found in Supplementary Table S7 and were obtained from the re-analysis of DAP-Seq data generated by O'Malley et al. (2016). The number of target promoters (Hit) containing the relevant CRE are depicted. Statistically significant CRE observations are represented with symbols *(0.01 > FDR > 1.0E–3), **(1.0E–3 > FDR > 1.0E–5), ***(FDR < 1.0E–17).

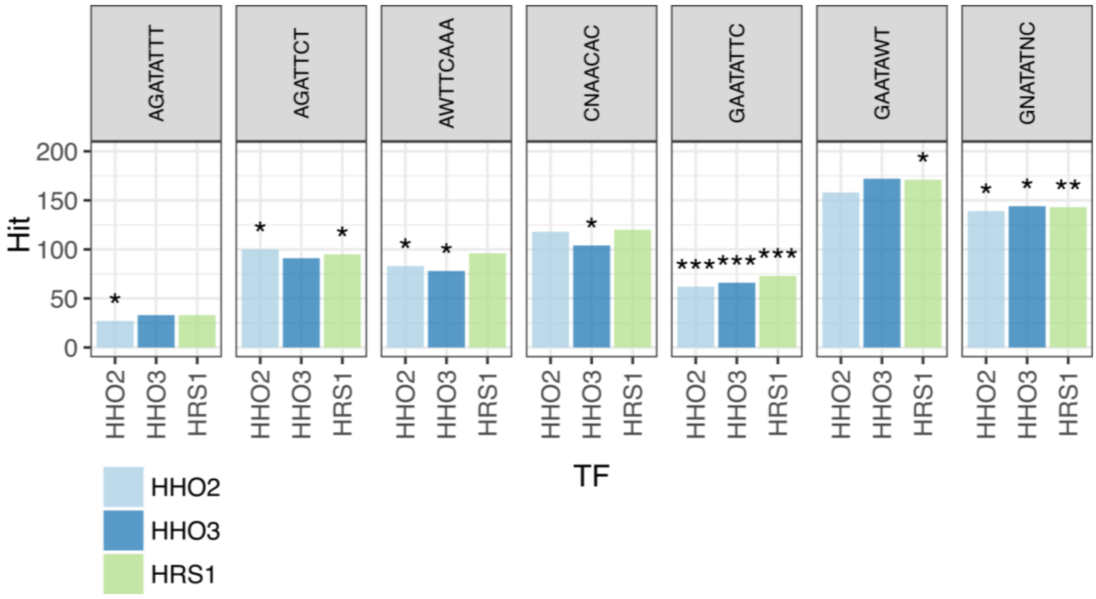

Fig. S5. Profiles of differential thermal analysis (DTA) for low temperature exotherms (LTEs) in Amur grape. (A) Empty vector (EV) control. (B-D) *VaAQUILO*-overexpression Amur grape calli lines (L1, L2, L3).

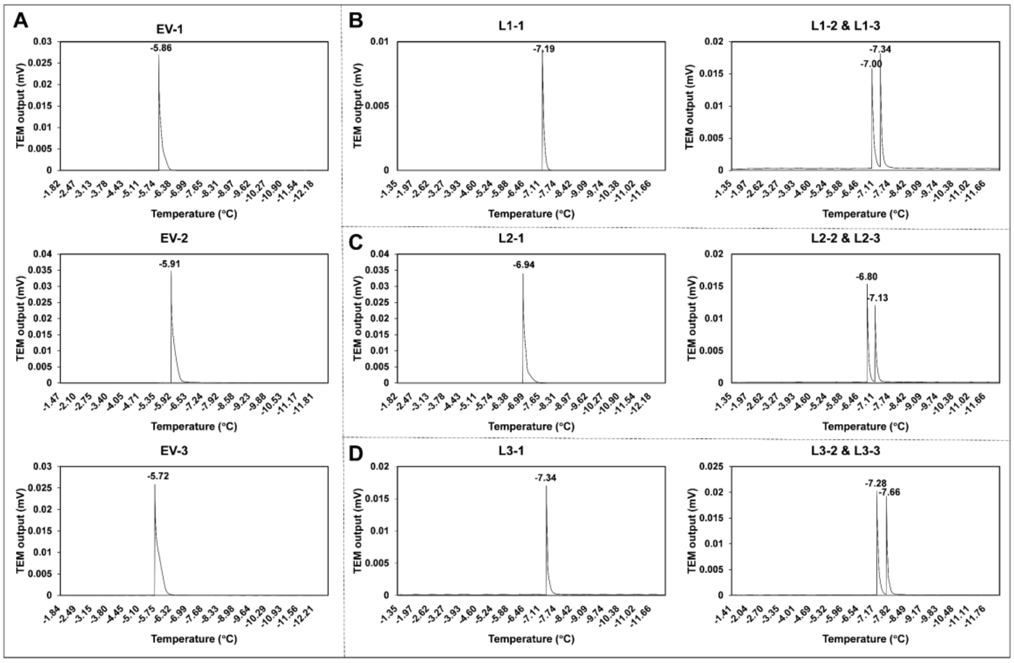

Fig. S6. Transcription factor binding motifs for the G2-type TFs AtHRS1, AtHHO2 and AtHHO3. Screen shot of web portal showing TF binding motif LOGOs obtained from DAP-Seq experiments generated by O'Malley et al. (2016): http://neomorph.salk.edu/dev/pages/shhuang/dap_web/pages/browse_table_aj.php. AGI: AtHRS1 (At1g13300), AtHHO2 (At1g68670) and AtHHO3 (At1g25550).

Protein	AGI	DAP/ampDAP	Reads	Peaks	FRiP	Top Motif
At1g13300	AT1G13300 [Araport]	DAP	1931754	883	3.16	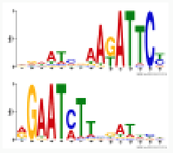
At1g68670	AT1G68670 [Araport]	DAP	3609244	9562	41.5	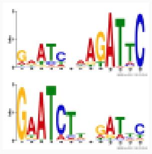
At1g25550	AT1G25550 [Araport]	DAP	3238016	11470	59.8	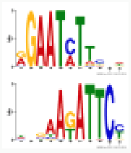

Fig. S7 (next page). Cold-induced galactinol and raffinose synthases in *Vitis*. (A) Phylogenetic relationships among Arabidopsis and grape galactinol and raffinose synthases. Maximum Likelihood (ML) phylogenetic tree based on 1000 iterations (bootstrap values are shown in decimals). Protein names were adopted following the nomenclature of Pillet *et al.* (2012). Additionally, letters differentiate proteins coded by genes in tandem in the same chromosome. Brackets refer to one-to-one or one-to-two cases of putative orthology. Blue labels in protein identifiers make reference to grape genes. Tree topology may vary as protein sequences are retrieved from gene model annotations present in the 12Xv1 and 12Xv2 genome accessions. (B) Expression responses of *AQUILO*, *GOLS* and *RAF* genes obtained from RNA-Seq data of *V. vinifera* cv. ‘Muscat Hamburg’ and *V. amurensis* cold-treated vegetative tissues (shoot apices with one well-developed leaf; Xin et al., unpublished data). Z-scored FPKM values are shown for cold-treated plantlets at 0, 2, 4, 8, 24 and 48 hours. C. Enzymatic pathway for the synthesis of raffinose family oligosacharids (RFOs). Enzymes in blue letters correspond to the proteins from the phylogenetic tree in (A).

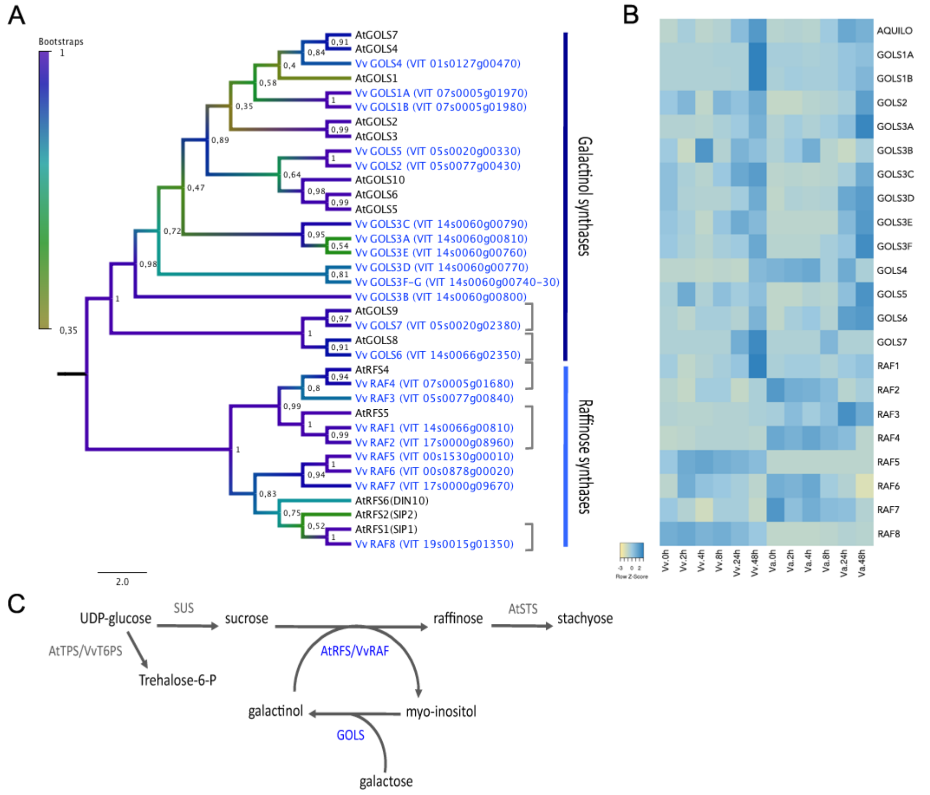

Fig. S8. Cold induction of Arabidopsis *GolS* and *RFS* genes. Publicly available Affymetrix (ATH1-0 chip) data consisted in 41 perturbation comparisons belonging to 157 samples. Hierarchical clusterization was performed with Euclidean distance algorithm. Data was processed in Genevestigator (Hruz *et al.*, 2008).

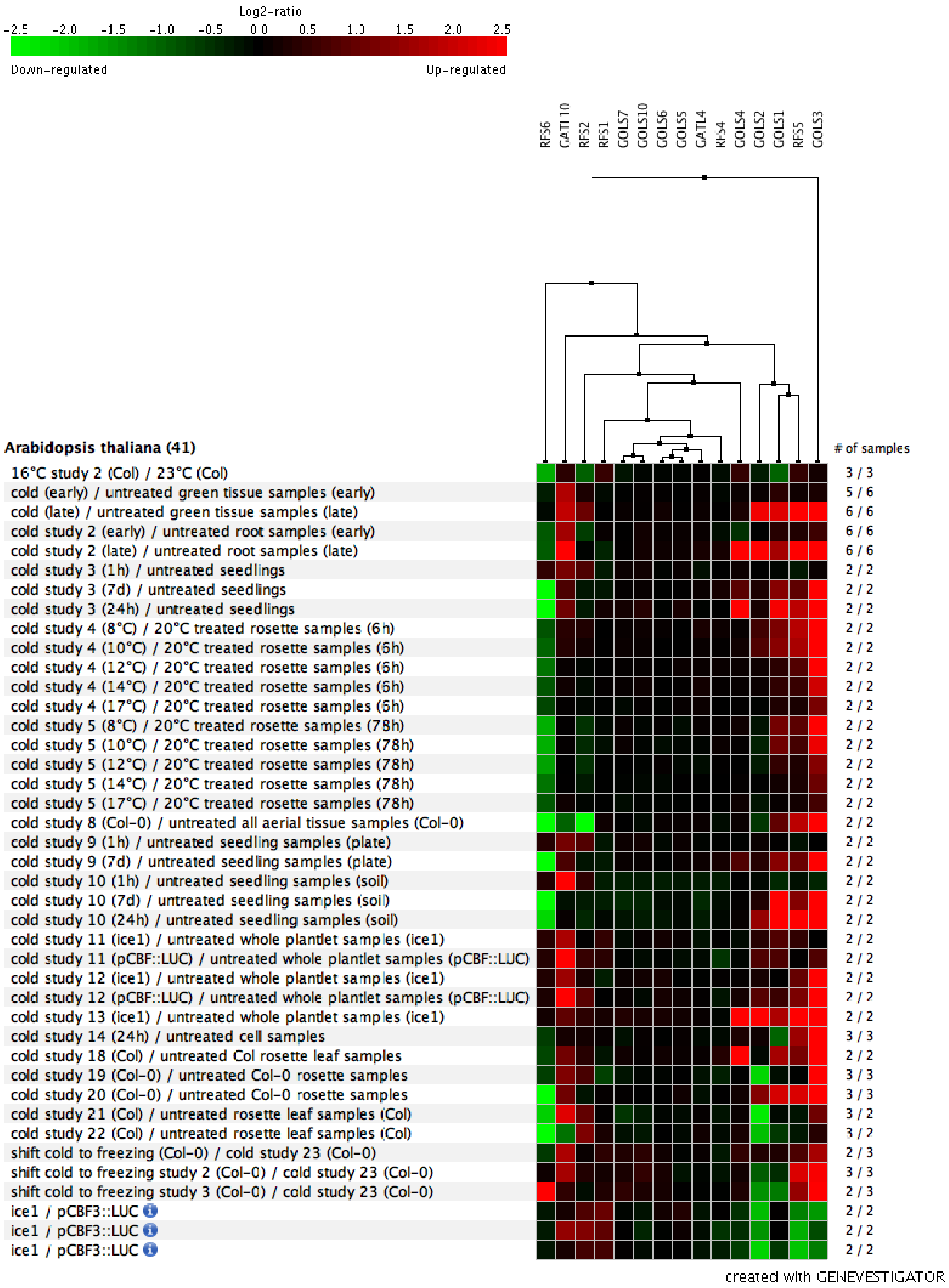

Fig. S9. Schematic representation of AQUILO-related processes identified in this work in relation with the cold response pathway.

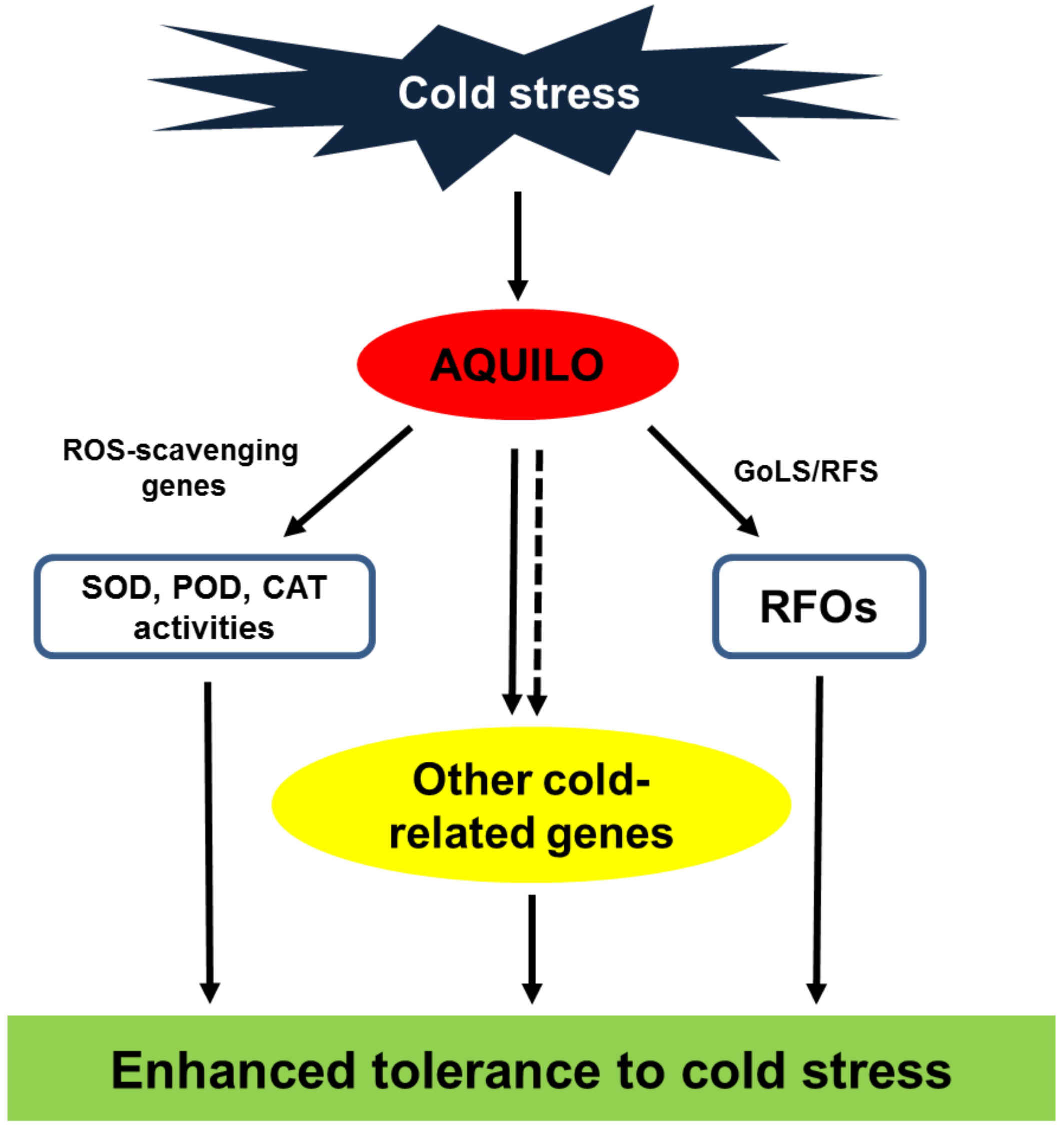

Fig. S10. Differential gene expression of *CBF1-4* in the *VaAQUILO* overexpressing Amur grape calli. *MALATE DEHYDROGENASE* (*MDH*, *VIT_07s0005g03350*) and *ACTIN* (*VIT_04s0044g00580*) genes were used as calibration qRT-PCR controls. Each reaction was performed in triplicate (technical replicates) with three independent biological replicates (n=3). The relative expression was calculated using the $2^{-\Delta\Delta Ct}$ method. Error bars represent standard errors (SE).

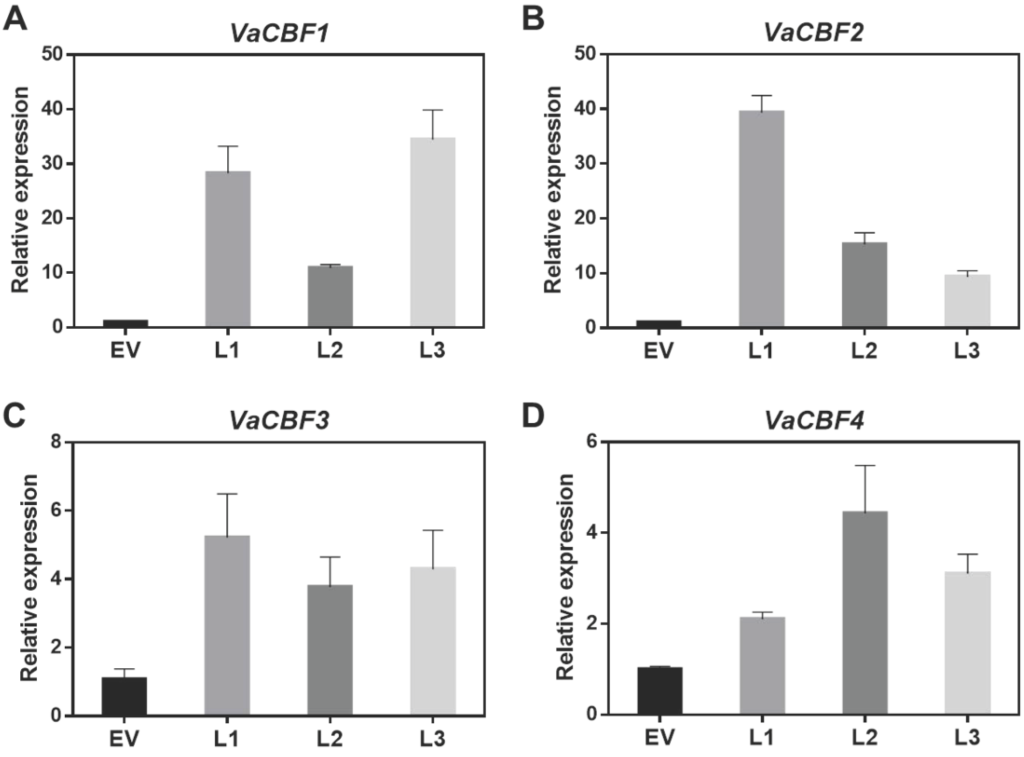

Supplement: Supplementary Figures S1-S10 [file ery020_suppl_supplementary_figures_s1-s10.pdf]
